# β-Herpesviruses in Febrile Children with Cancer

**DOI:** 10.3201/eid1404.070651

**Published:** 2008-04

**Authors:** Stephanie Yee-Guardino, Kate Gowans, Belinda Yen-Lieberman, Pamela Berk, Debra Kohn, Fu-Zhang Wang, Lara Danziger-Isakov, Camille Sabella, Sarah Worley, Philip E. Pellett, Johanna Goldfarb

**Affiliations:** * Cleveland Clinic Children’s Hospital, Cleveland, Ohio, USA; † Cleveland Clinic, Cleveland, Ohio, USA; 1 Current affiliation: University of North Carolina, Chapel Hill, North Carolina, USA; 2 Current affiliation: Wayne State University School of Medicine, Detroit, Michigan, USA

**Keywords:** Fever, neutropenia, cytomegalovirus, roseolovirus, human herpesvirus 6, human herpesvirus 7, pediatric cancer patients, research

## Abstract

We conducted a cross-sectional study of β-herpesviruses in febrile pediatric oncology patients (n = 30), with a reference group of febrile pediatric solid-organ transplant recipients (n = 9). One (3.3%) of 30 cancer patients and 3 (33%) of 9 organ recipients were PCR positive for cytomegalovirus. Four (13%) of 30 cancer patients and 3 (33%) of 9 transplant recipients had human herpesvirus 6B (HHV-6B) DNAemia, which was more common within 6 months of initiation of immune suppression (4 of 16 vs. 0 of 14 cancer patients; p = 0.050). HHV-6A and HHV-7 were not detected. No other cause was identified in children with HHV-6B or cytomegalovirus DNAemia. One HHV-6B–positive cancer patient had febrile disease with concomitant hepatitis. Other HHV-6B–positive children had mild “viral” illnesses, as did a child with primary cytomegalovirus infection. Cytomegalovirus and HHV-6B should be included in the differential diagnosis of febrile disease in children with cancer.

## Abstract 2

These viruses should be included in the differential diagnosis of febrile disease.

## CME ACTIVITY

Medscape, LLC is pleased to provide online continuing medical education (CME) for this journal article, allowing clinicians the opportunity to earn CME credit. Medscape, LLC is accredited by the Accreditation Council for Continuing Medical Education (ACCME) to provide CME for physicians. Medscape, LLC designates this educational activity for a maximum of 1.0 *AMA PRA Category 1 Credits*™. Physicians should only claim credit commensurate with the extent of their participation in the activity. All other clinicians completing this activity will be issued a certificate of participation. To participate in this journal CME activity: (1) review the learning objectives and author disclosures; (2) study the education content; (3) take the post-test and/or complete the evaluation at **http://www.medscape.com/cme/eid**; (4) view/print certificate.

## Learning Objectives

Upon completion of this activity, participants will be able to:

- Identify common infections associated with β-herpesviruses
- Specify β-herpesviruses isolated from children in the current study
- Describe clinical characteristics of β-herpesvirus infections in the current study
- List factors associated with higher rates of infection with human herpesvirus in the current study

## Editor

**D. Peter Drotman, MD**, Editor-in-Chief, Emerging Infectious Diseases. *Disclosure: D. Peter Drotman, MD, has disclosed no relevant financial relationships.*

## CME Author

**Charles P. Vega, MD**, Associate Professor; Residency Director, Department of Family Medicine, University of California, Irvine. *Disclosure: Charles P. Vega, MD, has disclosed that he has served as an advisor or consultant to Novartis, Inc*

## Authors

**Stephanie Yee-Guardino, DO**, Section of Pediatric Infectious Diseases, Cleveland Clinic Children’s Hospital, Cleveland, Ohio. *Disclosure: Stephanie Yee-Guardino, DO, has disclosed no relevant financial relationships.*

**Kate Gowans, MD**, Department of Pediatric Hematology and Oncology, Cleveland Clinic Children’s Hospital, Cleveland, Ohio.* Disclosure: Kate Gowans, MD, has disclosed no relevant financial relationships.*

**Belinda Yen-Lieberman, PhD**, Laboratory Medicine, Lerner Research Institute, Cleveland Clinic, Cleveland, Ohio. *Disclosure: Belinda Yen-Lieberman, PhD, has disclosed that she has received reagents at no charge from ARTUS Diagnostics.*

**Pamela Berk, BS**, Department of Molecular Genetics, Lerner Research Institute, Cleveland Clinic, Cleveland, Ohio. *Disclosure: Pamela Berk, BS, has disclosed no relevant financial relationships.*

**Debra Kohn, BS, MT**, Laboratory Medicine, Lerner Research Institute, Cleveland Clinic, Cleveland, Ohio. *Disclosure: Debra Kohn, BS, MT, has disclosed no relevant financial relationships.*

**Fu-Zhang Wang, PhD**, Department of Molecular Genetics, Lerner Research, Institute Cleveland Clinic, Cleveland, Ohio. *Disclosure: Fu-Zhang Wang, PhD, has disclosed no relevant financial relationships.*

**Lara Danziger-Isakov, MD, MPH**, Section of Pediatric Infectious Diseases, Cleveland Clinic Children’s Hospital, Cleveland, Ohio; Departments of Infectious Diseases, Lerner Research Institute, Cleveland Clinic, Cleveland, Ohio. *Disclosure: Lara Danziger-Isakov, MD, MPH, has disclosed no relevant financial relationships.*

**Camille Sabella, MD**, Section of Pediatric Infectious Diseases, Cleveland Clinic Children’s Hospital, Cleveland, Ohio; Departments of Infectious Diseases, Lerner Research Institute, Cleveland Clinic, Cleveland, Ohio. *Disclosure: Camille Sabella, MD, has disclosed that she has received grants from Merck, Inc. and Sanofi Pasteur.*

**Sarah Worley, MS**, Quantitative Health Sciences, Lerner Research Institute, Cleveland Clinic, Cleveland, Ohio. Disclosure: Sarah Worley, MS, has disclosed no relevant financial relationships.

**Philip E. Pellett, PhD**, Departments of Infectious Diseases, Department of Molecular Genetics, Lerner Research Institute, Cleveland Clinic, Cleveland, Ohio. Disclosure: Philip E. Pellett, PhD, has disclosed that he has received royalties from Chemicon International.

**Johanna Goldfarb, MD**, Section of Pediatric Infectious Diseases, Children's Hospital, Cleveland, Ohio; Departments of Infectious Diseases, Lerner Research Institute, Cleveland Clinic, Cleveland, Ohio. Disclosure: Johanna Goldfarb, MD, has disclosed no relevant financial relationships.*.*

## Earning CME Credit

To obtain credit, you should first read the journal article. After reading the article, you should be able to answer the following, related, multiple-choice questions. To complete the questions and earn continuing medical education (CME) credit, please go to **http://www.medscape.com/cme/eid**. Credit cannot be obtained for tests completed on paper, although you may use the worksheet below to keep a record of your answers. You must be a registered user on Medscape.com. If you are not registered on Medscape.com, please click on the New Users: Free Registration link on the left hand side of the website to register. Only one answer is correct for each question. Once you successfully answer all post-test questions you will be able to view and/or print your certificate. For questions regarding the content of this activity, contact the accredited provider, CME@medscape.net. For technical assistance, contact CME@webmd.net. American Medical Association’s Physician’s Recognition Award (AMA PRA) credits are accepted in the US as evidence of participation in CME activities. For further information on this award, please refer to http://www.ama-assn.org/ama/pub/category/2922.html. The AMA has determined that physicians not licensed in the US who participate in this CME activity are eligible for *AMA PRA Category 1 Credits*™. Through agreements that the AMA has made with agencies in some countries, AMA PRA credit is acceptable as evidence of participation in CME activities. If you are not licensed in the US and want to obtain an AMA PRA CME credit, please complete the questions online, print the certificate and present it to your national medical association.

## CME Questions

1. Which of the following statements about β-herpesviruses is *most* accurate?
  A. Infection is not common until late adolescence
  B. Infection with cytomegalovirus (CMV) promotes symptoms similar to mononucleosis
  C. Human herpesvirus (HHV)-6B is the cause of fifth disease
  D. HHV-7 is the virus responsible for most cases of roseola
2. Which of the following β-herpesviruses were detected in the patient cohort of the current study?
  A. HHV-6B and HHV-6A
  B. CMV and HHV-7
  C. HHV-6B and CMV
  D. HHV-6A and HHV-7
3. Which of the following statements about infection data in the current study is *most* accurate?
  A. The etiology of most patients’ fever was discovered during hospitalization
  B. Infection with β-herpesviruses occurred at a higher frequency among children with cancer compared with those with solid organ transplant
  C. Fever was generally higher among children infected with β-herpesviruses
  D. HHV-6B infection was likely a reactivation of previous infection among cancer patients
4. Which cancer factors promoted infection with HHV-6B in the current study?
  A. Solid organ tumor and over 6 months since the initiation of immune suppression
  B. Solid organ tumor and less than 6 months since the initiation of immune suppression
  C. Leukemia and less than 6 months since the initiation of immune suppression
  D. Leukemia and more than 6 months since the initiation of immune suppression

### Table. Activity Evaluation

**Table:** 

**1. The activity supported the learning objectives.**				
Strongly Disagree				Strongly Agree
1	2	3	4	5
**2. The material was organized clearly for learning to occur.**				
Strongly Disagree				Strongly Agree
1	2	3	4	5
**3. The content learned from this activity will impact my practice.**				
Strongly Disagree				Strongly Agree
1	2	3	4	5
**4. The activity was presented objectively and free of commercial bias.**				
Strongly Disagree				Strongly Agree
1	2	3	4	5

## β-Herpesviruses in Febrile Children with Cancer

Much remains to be learned about the pathogenic role of β-herpesviruses (cytomegalovirus [CMV], human herpesvirus 6 variants A and B [HHV-6A and HHV-6B], and human herpesvirus 7 [HHV-7]) in immune-compromised children. Most persons are infected with CMV, HHV-6B, and HHV-7 during childhood; the age of acquisition and clinical spectrum of HHV-6A have not been defined. In immune-competent children, CMV is associated with heterophile-negative mononucleosis, HHV-6B with roseola infantum (exanthem subitum or sixth disease), and HHV-7 with a small percentage of clinically recognized cases of roseola. However, most primary infections with these viruses are either asymptomatic or involve a nonspecific mild illness that can include fever, malaise, and abnormal liver function or hepatosplenomegaly (*1*–*4*). After primary infection, these viruses establish life-long residency in the host, seldom causing disease unless the immune system is weakened, as occurs after treatment for solid-organ and stem cell transplantation. In these patients, each of the β-herpesviruses can reactivate, manifesting as febrile and sometimes life-threatening illness including pneumonitis, encephalitis, bone marrow suppression, graft-versus-host disease, and organ rejection (*5*–*7*). In addition to having independent pathologic effects, β-herpesviruses may have additive or synergistic effects, as well as interactions with other infectious agents (e.g., fungal infections) (*8*,*9*).

Immune suppression caused by cancer treatment has many forms, often as pulses of cytotoxic agents that kill rapidly dividing cells, including lymphocytes. The risk for infections in pediatric cancer patients is well recognized, and much effort has been devoted to identifying and treating bacterial and fungal infections associated with fever and neutropenia (*10*–*14*). This effort usually involves hospitalization for empiric administration of intravenous antimicrobial drugs, even though most bacterial blood cultures remain negative; 40%–70% of such febrile episodes have no identifiable source (*15*,*16*). Some viral infections, such as those with herpes simplex or varicella zoster viruses, are associated with disease that can be serious and even fatal in pediatric oncology patients (*17*,*18*). Most episodes of fever are unexplained and assumed to be viral in nature (*19*).

Little attention has been paid to the possible contribution of β-herpesviruses as a cause of febrile illness in children with cancer, despite recognition that these viruses cause disease after organ transplantation. In studies that preceded application of PCR, CMV detection was associated with fever and hepatitis in children with malignancy (*20*,*21*). HHV-6 seroprevalence is similar in pediatric cancer patients and controls (*22*,*23*), but virus has been detected less frequently in saliva of children with cancer than that of healthy controls (*24*). In children from the Czech Republic, Michalek et al. detected both primary and reactivated HHV-6 and CMV infections during cytotoxic chemotherapy by using serologic analysis and PCR (*23*,*25*). Some HHV-6 infections were associated with severe disease, including pneumonitis, bone marrow aplasia, and persisting fever.

Because of the biologic plausibility of β-herpesvirus involvement in febrile illness in pediatric cancer patients and the paucity of PCR-era literature in this area, we conducted a cross-sectional study of the activity of these viruses in pediatric cancer patients and other immune-compromised children. The purpose of this study was to determine whether there is sufficient viral activity in these populations to warrant in-depth study and clinical consideration.

## Materials and Methods

### Patients

The study was reviewed and approved by the Cleveland Clinic Institutional Review Board. Informed consent was obtained from a parent or guardian of each person <18 years of age, or directly from persons >18 years of age; assent was obtained from children 7–17 years of age. Patients were enrolled from August 2004 through April 2005. Enrolled children were receiving treatment for a malignancy or were receiving immunosuppressive therapy after solid-organ transplantation (SOT). Inclusion criteria were an age of newborn to 21 years and new onset of fever with an oral or rectal temperature >38°C or an axillary temperature >37.5°C. At enrollment, we collected a blood specimen and information on age, sex, underlying illness and diagnosis, acute symptoms accompanying fever, and details about immune suppression (chemotherapy regimen, radiation therapy, immune modulators, and steroids). Gastrointestinal symptoms were defined as either vomiting or diarrhea. Clinical laboratory data collected included complete blood counts, renal and liver function test results, bacterial blood cultures, as well as results of testing conducted for evaluation of fever, such as respiratory virus analysis, stool studies, chest radiographs, and computed tomography scans.

### Specimens and Storage

Whole blood was collected in EDTA tubes and stored at 4°C until processed. A leukocyte pellet was collected from 1 mL of whole blood; 400 μL of plasma was preserved, 200 μL for PCR and 200 μL for serologic analysis. Pelleted leukocytes were added to MagnaPure LC lysis buffer (Roche Diagnostics, Indianapolis, IN, USA) and stored at −20°C.

### Virus Culture

CMV culture was conducted with pelleted leukocytes by using conventional MRC-5 cell shell vial cultures (Diagnostic Hybrids, Athens, OH, USA). Immune fluorescence testing was conducted after 48 hours by using a monoclonal antibody directed against the CMV immediate early protein (Chemicon, Temecula, CA, USA). Shell vial cultures were obtained for the first 11 patients enrolled; all cultures were negative. Because shell vial culture uses a large number of leukocytes and we were studying a neutropenic population, this procedure was discontinued to preserve cells for more sensitive PCR analysis (*26*). Purified lymphocytes were cultured to detect HHV-6 or HHV-7 infections as described (*27*,*28*). Cytopathic effect was recognized as enlarged and refractile cells, with confirmatory testing by PCR.

### Polymerase Chain Reaction

Template DNA was extracted by using the MagnaPure automated extraction method (Roche Diagnostics) and eluted into 50 μL of buffer. A total of 5 μL of eluted DNA was used in each PCR. The PCRs contained DNA extracted from the equivalent of ≈20 μL of plasma, leukocytes present in 100 μL of whole blood, and 20 μL of whole blood. For detection of CMV and HHV-6 variants A and B, the Artus CMV RG PCR ASR/Real Art/Qiagen RealArt CMV LC Assay and the RealArt/Qiagen HHV-6 A/B LC Assay (QIAGEN, Hamburg, Germany) were used. A co-amplified internal control was used to identify PCR inhibition. To differentiate between HHV-6 variants, the system uses the specific melting temperatures of the probes. Detection of HHV-7 was conducted by using previously described primers and hybridization probes (*29*). Real-time PCR was performed on a LightCycler system (Roche Diagnostics).

### Serologic Analysis

CMV serologic analysis was performed by using the VIDAS CMV IgG Assay (bioMérieux, Durham, NC, USA) in an automated instrument, according to the manufacturer’s protocol. Analyses for HHV-6 (using HHV-6A–infected cells) and HHV-7 were conducted by using conventional indirect immunofluorescence with commercial slides (Panbio Ltd, Columbia, MD, USA). Avidity of antibody to HHV-6 was tested as described by Ward et al. (*30*).

### Statistical Analysis and Clinical Associations

The exact unconditional test was used to compare groups on viral activity (*31*). All tests were 2-tailed and performed at a significance level of 0.05. Analyses were performed by using StatXact PROCs version 6.3 (Cytel Software Corp., Cambridge, MA, USA) and SAS version 9.1 (SAS Institute Inc., Cary, NC, USA). Clinical associations with β-herpesvirus infection were based on symptoms and concomitant laboratory findings in patients who had no other identifiable cause for their febrile illness.

## Results

### Patient Characteristics

Forty-one children were enrolled, from whom 39 evaluable specimens were available (Table 1). Thirty of the 39 children had a malignancy (our primary interest) and 9 were organ transplant recipients who were included for comparison. All cancer patients were receiving active therapy, except for 1 child who was included in the study because she had been receiving chemotherapy for 2.5 years, which ended 3 months before study enrollment. Several patients had symptoms in addition to fever, mostly upper respiratory or gastrointestinal. A source of fever was identified for 5 (17%) of 30 cancer patients and 2 (22%) of 9 organ transplant recipients. Two cancer patients had bacteremia (*Escherichia coli*, coagulase-negative staphylococci); no fungal infections were detected. Three cancer patients and 2 organ transplant recipients had respiratory viral infections diagnosed by symptoms and positive direct fluorescent antibody test results. Of the 39 children, levels of liver enzymes were obtained for 28 as part of routine care. Of these children, 6 had abnormal values; 2 of these 6 also had virus detected (HHV-6B in 1 patient and influenza A in the other). The other 4 patients with increased levels of liver enzymes had longstanding abnormalities; review of their records suggested that the abnormal results were caused by underlying disease in 1 liver transplant patient and chemotherapy in 3 cancer patients. Six patients have died since completion of our study, none from infectious causes.

**Table 1:** Characteristics of febrile immune-suppressed children*

Characteristic	Cancer (n = 30)	SOT (n = 9)
Age, y; mean, median, range	6.1, 5.0, 0.4–17	11.8, 13.3, 4.3–20.6
Sex		
F	14	7
M	16	2
Basis of immune suppression		
Cancer	30 (77%)	–
Solid tumor	20	–
Leukemia	9	–
Lymphoma	1	–
Solid-organ transplant	–	9 (23%)
Heart	–	5
Lung	–	2
Liver	–	2
Immune suppressants	29	9
Cytotoxic chemotherapy	28	–
Radiation	6	–
Chemotherapy and radiation	6	–
Immune modulators†	–	9
Steroids (past 6 months)	14	7
Signs and symptoms		
Ill appearance	7	1
Upper respiratory	17	1
Gastrointestinal	10	7
Headache	3	3
Rash	0	0
Seizure	0	0
Hepatitis	1	1
Blood products (past 6 months)‡	20	5
Laboratory parameters		
Neutropenic (<500 cells/μL)	14	2
Lymphopenic (<500 cells/μL)	13	4
Established source for fever		
Bacteremia	2	0
Positive respiratory DFA test result§	3	2

*SOT, solid-organ transplantation; –, not applicable; DFA, direct fluorescence antigen. †Immune modulators included tacrolimus in 4, tacrolimus and azathioprine in 2, cyclosporine and mycophenolate mofetil in 2, sirolimus in 1, and tacrolimus, mycophenolate mofetil, antithymocyte globulin (rabbit), and corticosteroids in 1. ‡Blood products recorded included packed erythrocytes or platelets. §Of the 5 patients with positive respiratory DFA test results, 2 were positive for parainfluenza 3, 2 for influenza A, and 1 for respiratory syncytial virus.

### Laboratory Results

Of the 11 specimens tested in the shell vial assay, none was positive for CMV. CMV DNA was detected by PCR in lymphocytes of 1 (3.3%) of 30 cancer patients and 3 (33%) of 9 organ transplant recipients (Table 2).

**Table 2:** Detection of β-herpesviruses in cancer patients and solid-organ transplant recipients*

Characteristic	Cancer patients, no. positive/no. tested (%)		Transplant patients (n = 9), no. positive/no. tested (%)
	Leukemia (n = 9)	Solid tumor (n = 20)	
CMV PCR			
Whole blood	1/6 (17)	0/5	3/7 (43)
PBMC	1/7 (14)	0/16	2/8 (25)
Plasma	0/9	0/20	2/9 (22)
All tests	1/9 (11)	0/20 (0)	3/9 (33)
HHV-6 PCR			
Whole blood	2/6 (33)	0/5	2/7 (29)
PBMC	1/7 (14)	1/16 (6)	3/8 (38)
Plasma	0/9	0/19	1/9 (11)
Lymphocyte culture	1/8 (13)	0/16	0/9
All tests	3/9 (33)	1/20 (5)	3/9 (33)
HHV-7 PCR			
Whole blood	0/6	0/5	0/7
PBMC	0/7	0/16	0/8
Plasma	0/9	0/20	0/9
All tests	0/9	0/20	0/9

*Data are not shown for study participant 23, a patient with lymphoma who did not have β-herpesviruses detected in whole blood, PBMC, or plasma. CMV, cytomegalovirus; PBMC, peripheral blood mononuclear cells; HHV-6, human herpesvirus 6.

Lymphocyte cultures for HHV-6 and HHV-7 were obtained for 34 of the 39 children. Of these, 15 cultures showed cytopathic effects similar to those commonly reported for HHV-6, but none of these were positive for HHV-6 by PCR. Cultured cells from 1 patient with leukemia (study participant 19) were positive for HHV-6B by PCR, in the absence of a cytopathic effect. Four (13%) of 30 cancer patients and 3 (33%) of 9 transplant recipients (p = 0.19) had HHV-6B DNAemia; HHV-6A was not detected. Viral loads ranged from 50 to 500,000 DNA copies/mL. Of 7 PCR-positive patients, 3 also showed positive results in peripheral blood mononuclear cells (PBMC) and whole blood specimens, 2 in only PBMC, and 1 only in a lymphocyte culture. Patient 40, a transplant recipient, had positive results in all specimens tested including PBMC, whole blood, and plasma, with the highest viral load (>5 × 10^5^ genomes/mL) in PBMC. These results are consistent with either severe acute infection or the child having germline integrated HHV-6B (*32*,*33*). PCR positivity was more common in children whose blood was collected within 6 months of initiation of immune suppression compared with children sampled >6 months after initiation of immune suppression (cancer patients: 4 of 16 vs. 0 of 14 after 6 months, p = 0.050; transplant recipients: 2 of 3 vs. 1 of 6, p = 0.26) (Figure). Solid-tumor patients were less likely to be positive for HHV-6B than either leukemic (1 of 20 vs. 3 of 9, p = 0.046) or SOT patients (1 of 20 vs. 3 of 9, p = 0.046). Two of the HHV-6B-positive SOT patients had concurrent CMV DNAemia.

**Figure:**
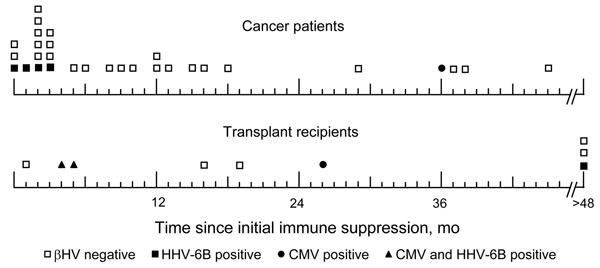
Temporal relationship between detection of β-herpesvirus (βHV) and onset of immune suppression. Time of collection of each study specimen is indicated relative to onset of immune suppression for cancer patients and solid-organ transplant recipients. HHV, human herpesvirus; CMV, cytomegalovirus.

HHV-7 DNA was not detected in any patients. Lymphocyte cultures were not tested for HHV-7 by PCR because none of the plasma, whole blood, or leukocyte pellets were positive for the virus.

### Possible Associations between β-Herpesvirus Activity and Disease

Patients who were positive for β-herpesviruses and those who were negative for these viruses did not differ in their average fever temperature (38.5°C vs. 38.6°C). In SOT recipients, the prevalence of HHV-6B and CMV and the spectrum of their associated illnesses were concordant with prior reports. Thus, we will focus on the cancer patients.

The only cancer patient with CMV DNAemia appeared to have primary CMV infection on the basis of negative results for immunoglobulin (Ig) G to CMV, negative bacterial blood culture, and age ( Appendix Table). This patient was a 5.4-year-old child (study participant 29) with acute lymphoblastic leukemia (ALL) in remission; he had upper respiratory symptoms and fever but otherwise appeared well. He was treated with an antimicrobial drug (ceftriaxone) on an outpatient basis, but his blood culture was negative for bacteria and no other source for his fever and illness was found. This child began cancer chemotherapy 36 months before the febrile event.

Four patients appeared to have reactivated HHV-6B infection on the basis of positive results for virus DNA, high-avidity IgG, and clinical symptoms. The first patient (study participant 19) was a 2-year-old boy with ALL in remission for the first time. In the second month of chemotherapy; he was hospitalized for fever and ill appearance, but he was not neutropenic or lymphopenic. He had marked hepatomegaly (≈10 cm below the right costal margin) and abnormal results in liver function tests. He was receiving fluconazole for oral thrush, and his liver enzyme levels were mildly increased before the febrile illness. However, at admission he had an aspartate aminotransferase (AST) level of 2,356 U/L and an alanine aminotransferase (ALT) level of 2,410 U/L. HHV-6 and HHV-7 IgM and IgG titers were obtained (Specialty Laboratories, Valencia, CA, USA) as part of his ALL treatment protocol. His HHV-6 IgM titer was negative (<40), and his IgG titer was positive (320) and high-avidity antibodies were detected. Results for HHV-7 IgM were negative, and those for IgG were weakly positive (titer 20). Other studies conducted by the primary medical team included tests for acute hepatitis and a CMV PCR; all results were negative. Four days later, his AST level was 84 U/L and his ALT level was 314 U/L. On follow up 7 days after admission, both values were near normal. The hepatitis was attributed to methotrexate, which was therefore withheld until the levels of liver enzymes returned to normal. On the basis of the positive culture for HHV-6B and hepatitis in the setting of febrile illness without other identified infections, we suspect that reactivated HHV-6B infection caused the fever and exacerbated the hepatitis.

A 5-year-old girl with relapsed acute myeloid leukemia (study participant 23) was intermittently febrile while hospitalized for 1 month during chemotherapy. She was treated with broad-spectrum antibacterial drugs and antifungal drugs. At study enrollment on day 12 of hospitalization, she had an absolute neutrophil count of 0 and an absolute lymphocyte count of 560 cells/μL. Daily blood cultures and sporadic respiratory viral studies did not reveal a cause for her fever, but her whole blood and PBMC were positive for HHV-6B by PCR.

HHV-6B was detected in a 2-year-old girl with ALL in remission (study participant 25) and in a 22-month-old boy with stage 3 Wilms tumor (study participant 38). Both of these patients were lymphopenic and had nonspecific febrile illnesses; neither had other identified sources of fever.

We suspect primary CMV infection in 1 patient and reactivated HHV-6B infection in 4 cancer patients as likely causes of the febrile episodes. This conclusion is based on a combination of clinical data, positive PCR findings, and lack of other identified sources for fever.

## Discussion

β-Herpesviruses are known pathogens in immunocompromised patients, but they have not been extensively studied in children with malignancies. In this study of children who had fever, signs of infection, and had samples taken at 1 time point, we detected β-herpesviruses in 5 of 25 cancer patients who had no other source of fever identified: 4 had HHV-6B DNA and 1 had CMV DNA detected by PCR. β-Herpesviruses were detected in 4 of 7 SOT patients who had no other source of fever identified: 1 had HHV-6B, 1 had CMV, and 2 had both virues (Figure). One of these children was ill and had a high fever and pleuritis with no other pathogens identified. HHV-6A and HHV-7 were not detected in any of the cancer or SOT patients. In our study, frequencies of HHV-6B were similar in transplant recipients and cancer patients (3 of 9 vs. 4 of 30, p = 0.19).

HHV-6B was detected more frequently in children with leukemia and solid-organ transplants than in children with other cancers. This finding may be the result of similarities in the degree and type of immune suppression. Children with leukemia and SOT patients generally receive intense initial immune suppression followed by a constant lower level of suppression. In contrast, solid-tumor patients generally receive monthly immune-suppressive treatments and seldom have prolonged lymphopenia. HHV-6B DNAemia was more frequent in children sampled during the first 6 months of immune suppression when immune suppression is most intense, which is consistent with findings for pediatric bone marrow and solid-organ transplant recipients (*34*). Detection of HHV-6B early after initiation of therapy in patients with cancer (Figure) suggests that the presence of virus DNA may be related to intense immune suppression and not to random occurrence, which would not have clustered early in therapy.

Our observations of possible clinical associations with β-herpesvirus infection concur with those of Michalek et al. (*23*,*25*) of a cohort dating from 1993 to 1999. Our population included 1 child with suspected HHV-6 disease and hepatitis, which has been seen in association with HHV-6 activity after liver (*35*), renal (*36*), and stem cell transplantation (*37*).

In contrast to laboratory detection for CMV, testing for HHV-6 and HHV-7 DNA is often conducted by using in-house assays, which makes comparison across studies difficult. We used an assay for detecting HHV-6 DNA that is commercially available in Europe. This test was concordant with our in-house assay in detecting previously characterized HHV-6 isolates (D. Kohn et al., unpub. data). In addition to standardization of molecular testing, various methods for detecting HHV-6 during febrile episodes should be evaluated because some investigators have suggested that viral isolation by culture may be more useful in correlating active infection with clinical disease (*38*). Furthermore, reverse transcription–PCR can distinguish active replication from latency (*39**,**40*).

Several limitations are inherent to our cross-sectional study design. First, capturing data at single time points limits the ability to assess the temporality of the virologic burden in relation to disease. Second, although we found HHV-6B positivity during febrile events in 18% of our population, we do not have data during periods without fever and therefore cannot be certain that viremia is specifically associated with disease. The studies by Michalek et al. also included treatment-phase blood specimens that were obtained only during febrile illness or other clinical events suggestive of viral infection (*23*,*25*). Thus, activity of HHV-6 and other lymphotropic viruses during nonfebrile periods in this patient population remains unknown. We studied a heterogeneous patient population that included several types of cancer; the level of immune suppression varied widely. Finally, identification of possible causes of febrile episodes was limited by the assays we performed and the tests ordered by the primary medical team. Thus, we cannot exclude other viral pathogens such as human metapneumovirus or Epstein-Barr virus for which no testing was conducted.

Evidence for our suspected associations between HHV-6B and the febrile events includes 1) our use of relatively small volumes of blood in the PCR analyses, which reduces the chance of detecting sporadic latently infected lymphocytes; 2) the temporal association of detection of HHV-6B with initiation of chemotherapy; and 3) a lack of other identified sources for the fever. With the exception of the transplant recipient with possible HHV-6B germline integration, HHV-6B or CMV are plausible causes of the febrile episode in every instance in which they were detected.

Our study differs from prior studies in that we assayed for all 4 β-herpesviruses and considered the timing of viral activity relative to the onset of immune suppression. We included solid-organ transplant patients as a reference for cancer patients, and excluded stem cell transplant patients, a population in whom reactivation of CMV and HHV-6 has been studied. By enrolling children with a variety of malignancies, we surveyed a diverse cancer population.

Although the number of patients we studied was small, CMV and HHV-6B are clearly active in pediatric cancer patients. The lack of other identified agents coincident with fever suggests that these viruses may cause febrile illness in this population. Our observations, combined with those of Michalek et al. (*23*,*25*), suggest that HHV-6B and CMV should be included in the differential diagnoses of febrile disease in children with cancer. Further investigation in this patient population is needed to clarify the role of β-herpesviruses in these febrile illnesses.

## Supplementary Material

Appendix Table Characteristics of cancer patients with suspected ?-herpesvirus infection on the basis of DNAemia and clinical signs and symptoms*
