# Supplementary material for: β-Herpesviruses in Febrile Children with Cancer
Source: Emerg Infect Dis. 2008 Apr;14(4):579–85. doi: 10.3201/eid1404.070651 (PMC11323659; doi:10.3201/eid1404.070651)
Supplement: Appendix Table — Characteristics of cancer patients with suspected ?-herpesvirus infection on the basis of DNAemia and clinical signs and symptoms* [file 07-0651_appT-s1.pdf]

Appendix Table. Characteristics of cancer patients with suspected β-herpesvirus infection on the basis of DNAemia and clinical signs and symptoms*

Study no.	Interval, mo†	Clinical status	Age, y	Chemo-therapeutics	Symptoms in addition to fever	Fever duration, d	Pertinent physical findings	General appearance	AV	Pertinent laboratory values	Viral load (copies/mL)							
											HHV-6B				CMV			
											WB	PBMC	PL	LC	WB	PBMC	PL	LC
23	<1	AML recurrence‡	5	ARA-C, gemtuzumab, ozogamicin	Diarrhea	6	None	Well	None	ANC 0, ALC 560, high-avidity antibody to HHV-6	1,560	495	–	–	–	–	–	–
19	1	ALL in remission	2.1	Asparaginase, 6-MP, MTX, vincristine, decadron	URI	1	Hepatomegaly, red left tympanic membrane	Ill	None	AST 2,356, ALT 2,410, high-avidity antibody to HHV-6	NT	NT	–	685	NT	NT	–	–
25	2	ALL in remission	2.3	Daunorubicin, MTX, vincristine, ARA-C	None	1	None	Well	None	ANC 550, high-avidity antibody to HHV-6	58	–	–	NT	–	–	–	–
38	3	Wilms tumor	1.9	Actinomycin-D, vincristine	None	1	None	Well	None	ALC 530, high-avidity antibody to HHV-6	NT	97	–	NT	NT	–	–	NT
29	36	ALL in remission	5.4	6-MP, MTX	URI	1	None	Well	None	ALC 300, CMV IgG negative	–	–	–	–	26,300	25	–	–

*AV, antiviral therapy; HHV-6B, human herpesvirus 6B; CMV, cytomegalovirus; WB, whole blood; PMBC, peripheral blood mononuclear cells; PL, plasma; LC, lymphocyte culture, AML, acute myeloid leukemia; ARA-C = cytarabine, ANC, absolute neutrophil count in cells/μL; ALC, absolute lymphocyte count in cells/μL; ALL, acute lymphphblastic leukemia; 6-MP, 6-mercaptopurine; MTX, methotrexate; URI, upper respiratory infection; AST, aspartate aminotransferase in U/L; ALT, alanine aminotransferase in U/L; NT, not tested (i.e., specimen not available or equipment failure); IgG, immunoglobulin G.

†Time between fever and onset of immune suppression.

‡We describe this patient as immune suppressed for <1 mo. This patient originally was treated for AML and had ended therapy 12 months before study enrollment.
